# A Novel miRNA Restores the Chemosensitivity of AML Cells Through Targeting FosB

**DOI:** 10.3389/fmed.2020.582923

**Published:** 2020-10-06

**Authors:** Huiwen Wang, Huien Zhan, Xinya Jiang, Lilian Jin, Tianming Zhao, Shurong Xie, Wei Liu, Yan Jia, Hui Liang, Hui Zeng

**Affiliations:** ^1^Department of Hematology, Xiangya Hospital, Central South University, Changsha, China; ^2^Department of Hematology, The First Affiliated Hospital of Jinan University, Guangzhou, China

**Keywords:** acute myeloid leukemia, FosB, chemosensitivity, miRNA, targeting therapy

## Abstract

The heterogeneous nature of acute myeloid leukemia (AML) and its poor prognosis necessitate therapeutic improvement. Current advances in AML research yield important insights regarding both AML genetics and epigenetics. MicroRNAs (miRNAs) play important roles in cell proliferation, differentiation, and survival and may be useful for AML diagnosis and prognosis. In this study, a novel miRNA, hsa-miR-12462, was identified in bone marrow (BM) samples from AML patients at diagnosis by small RNA sequencing. A significant higher level of hsa-miR-12462 was found in patients who achieve complete remission (AML-CR) after induction therapy compared with those who suffer relapse/refractory (AML-RR). FosB was predicted to be the target of hsa-miR-12462 through RNA sequencing, bioinformatics analysis, and protein–protein interaction (PPI) network analysis and then verified by luciferase activity assay. T-5224, the inhibitor of FosB, was administered to AML cell lines, which could inhibit cell proliferation, promote apoptosis, and restore the sensitivity of AML cells to cytarabine (Ara-C). In summary, a higher level of hsa-miR-12462 in AML cells is associated with increased sensitivity to Ara-C *via* targeting FosB.

## Introduction

Acute myeloid leukemia (AML) is a highly heterogeneous hematologic malignancy characterized by unlimited clonal proliferation of myeloid blasts. In the United States, almost 20,000 new patients suffer from AML and up to 11,000 patients die from the disease per year (1). The standard treatment is induction therapy followed by consolidation in the manner of chemotherapy or hematopoietic stem cell transplantation (HSCT), aiming to eradicate the residual disease (2). Since the 1970s, the mainstay of induction therapy consisting of cytarabine (Ara-C) with anthracycline has been established and rendered relatively favorable complete remission (CR) rates. However, high recurrence rate and drug resistance remain a Gordian knot to further improve the patients' survival. Therefore, exploring elaborate mechanisms and exploiting novel agents are consistently emphasized. From 2017 to 2018, at least eight new drugs have been approved by Food and Drug Administration (FDA) for the treatment of AML. The dramatic emergence in epigenetic knowledge is somewhat responsible for part of these advances. For example, hypomethylating agents (decitabine and azacitidine) have been widely applied for elderly patients, and a novel histone deacetylase inhibitor chidamide has also been approved by China SFDA in the near past. Thus, focusing on epigenetic alternations might be promising in targeting therapy of AML.

MicroRNAs (miRNAs) are a class of endogenous small non-coding RNAs, each with a single strand of 22 nucleotides approximately (3). By binding to partially complementary sites of the 3′-untranslated regions (3′-UTRs) of specific target mRNA, miRNAs regulate the gene expression at the posttranscriptional level *via* degrading mRNA or inhibiting their translation (4). The aberrant expression of miRNAs was proved to be closely related to the occurrence and development of tumor, including proliferation, differentiation, and apoptosis (5), which enabled clinicians to predict the disease status and prognosis (6).

The activator protein (AP-1) is a ubiquitously expressed dimeric transcription factor complex comprising proteins belonging to the Jun, Fos, activating transcription factor (ATF), or MAF BZIP transcription factor (MAF) protein families (7). As a pivotal role, AP-1 modulates the transcription of multiple cytokines and growth factors and is implicated in the proliferation, survival, differentiation, and transformation of cells (8). Regarding hematologic malignancies, abnormalities of AP-1 components exist in AML (9), chronic myelogenous leukemia (CML) (10), Hodgkin's disease (HD) (11), as well as anaplastic large cell lymphoma (ALCL) (12). Staber et al. (9) claimed that the expression of c-Jun and c-Fos in the transcriptional level was enhanced in relapsed AML over untreated patients, indicating that the AP-1 family is associated with poor prognosis, while the mechanism of other AP-1 components, such as FosB, has not been investigated in AML. In pancreatic cancer, miR-144-3p plays an important role in migration and invasion by targeting FosB (13). Thus, it is intriguing whether the interaction between miRNA and FosB participates in regulating the phenotypes of AML.

In our research, a novel miRNA, hsa-miR-12462, was determined by small RNA sequencing and stood out from the rest in the AML-CR group. Our latest study has verified that overexpression of hsa-miR-12462 inhibits the growth of AML cells and enhances Ara-C sensitivity (14). Next, we aimed to explore the potential target of hsa-miR-12462 and its contribution in maintaining indefinite proliferation and intractable drug sensitivity as to AML cell lines.

## Materials and Methods

### Patient Enrollment

A total of 128 bone marrow (BM) specimens from AML patients aged 15–71 years old were collected at the outpatient and inpatient departments from January 2016 to November 2019. All specimens were collected before chemotherapy and grouped into cohort groups based on curative effect: (1) those achieving a CR with conventional induction chemotherapy and remaining in CR ≥6 months (AML-CR cohort); and (2) those not achieving CR after two courses of standard induction chemotherapy (refractory) or relapsed in <6 months after CR (relapsed) (AML-RR cohort). All specimens were collected after chemotherapy and divided into two cohorts based on curative effect, referring to the AML-RR cohort and the AML-CR cohort. The group criteria are consistent with the above. All BM samples were collected into sterile tubes containing anticoagulant (heparin sodium). Mononuclear cells (MNCs) were enriched by density centrifugation with Ficoll-Paque (Sigma, St. Louis, MO, USA) and stored at −80°C. The study protocols were approved by the Medical Institutional Ethics Committee of the Xiangya Hospital of Central South University. Written informed consent was obtained from all participants. Diagnosis of AML was carried out according to the 2016 WHO criteria.

### Cell Culture and Lentivirus Transfection

Three human AML cell lines and two normal tissue cell strains were used in this study. All cell lines were obtained from the Cell Resource Center (Xiangya Medical College, Central South University, Hunan, China). U937, THP-1, and HL-60 cell lines were cultured in RPMI-1640 medium (Hyclone, USA) supplemented with 10% fetal bovine serum (Solarbio, Beijing, China) and 1% antibiotic solution of penicillin and streptomycin (Hyclone, USA). 293T and human umbilical vein endothelial cells (HUV-EC-C) cell lines were cultured in Dulbecco's modified Eagle's medium with high glucose (DMEM) (General Electric, USA) containing 10% fetal bovine serum (Solarbio, Beijing, China). Cells were incubated in a humidified atmosphere containing 5% CO_2_ at 37°C. To overexpress hsa-miR-12462, U937, THP-1, and HL-60 were infected with lentivirus (lentivirus vector: pGC-FU-3FLAG-SV40-EGFP-IRES-puromycin; promoter: Ubiqutin) containing the open reading frame of hsa-miR-12462 [multiplicity of infection (MOI): [30]]. After 12 h of infection, we replaced the media for the cell lines and continued to culture for another 72 h. Then, the transfection efficiency was observed under a fluorescence microscope.

### Dual Luciferase Reporter Assay

293T cells were inoculated at a density of 50% into a 96-well plate and transfected with luciferase reporter plasmids after 16 h when reaching a confluency of 70%. Each sample set was placed in three multiple wells. First, four groups were designed: blank group (transfection reagent + cells), plasmid group, plasmid + hsa-miR-12462 NC group, and plasmid + hsa-miR-12462 mimic group. After co-transfection for 18–48 h, Firefly luciferase and Renilla luciferase activity were detected by the dual luciferase reporting analysis system. Each sample had two values: RLU1-Firefly luciferase reaction intensity and RLU2-inner Renilla luciferase reaction intensity, and the ratio of the two groups was calculated, namely, RLU1/RLU2.

### Quantitative RT-PCR for microRNA and mRNA Expression

Total RNA (500 ng) was isolated from 1 × 10^6^ cells using Trizol reagent (Promega, USA). For hsa-miR-12462, Bulge-LoopTM miRNA RT primer was obtained from RiboBio Corporation (Guangzhou, China). miDETECT A Track miRNA qRT-PCR Starter Kit (RiboBio, China) was applied according to the manufacturer's instructions. For FosB, All-in-One First-Strand cDNA Synthesis Kit (GeneCopoeia, Rockville, USA) was applied to synthesize cDNA, and qPCR assay was carried out using Hieff qPCR SYBR^®^ Green Master Mix kit (Yeasen Biotech, Shanghai, China) with an Applied Biosystems Prism machine using ABI StepOnePlus (Applied Biosystems, Foster City, CA, USA). The primers of FosB were designed by GeneCopoeia Inc., China. The relative expression level of hsa-miR-12462 and FosB was normalized to U6 and glyceraldehyde 3-phosphate dehydrogenase (GAPDH), respectively, and calculated using the 2-ΔΔCT method.

### Western Blotting

Equal amounts of protein were separated by 10% sodium dodecyl sulfate–polyacrylamide gel electrophoresis (SDS-PAGE) and then transferred electrophoretically to polyvinylidene fluoride (PVDF) membranes (Millipore, Billerica, MA, USA). The membranes were incubated with TBST containing 5% bovine serum albumin (BSA) (Bio Sharp Sigma A-4612) for an hour at room temperature and then with primary antibodies overnight at 4°C. After incubation with secondary antibodies, the protein bands were detected with a ChemiDoc TMMP imaging system (Bio-Rad Laboratories. Inc., Hercules, CA, USA).

### Cytotoxicity Assay

To evaluate the cell response to different drugs, cell proliferation was determined by the Cell Counting Kit-8 (7sea biotech, China) assay after treatment. In brief, HL-60 and THP-1 cells (5 × 10^4^/ml) were seeded in 96-well culture plates and incubated with T-5224 (Selleck, USA) (concentration gradient is set to 0, 10, 20, 40, 60, and 80 μM) or united Ara-C (SinoPharm YiXin, China) (concentration gradient is set to 0.625, 1.25, 2.5, 5, 10, and 20 μM) treatment for a period of time (24, 48, and 72 h). After certain times, 10 μl of CCK8 solution was added to each well for a 3-h culture at 37°C. Absorbance was measured by a spectrophotometer (Bio Tek Instruments, USA) at a wavelength of 450 and 630 nm. The calculation formula of relative cell vitality (%) is: (experimental well - blank well)/(control well - blank well) × 100%.

### Apoptosis Assessment

A total of 5 × 10^6^ cells were inoculated in six-well culture plates and treated with T-5224 at final concentrations of 0, 40, and 80 μM, respectively. After 24 h incubation, cells were collected and treated with Annexin V-7-AAD kit (Multi Sciences, China) and subjected to flow cytometry (Becton Dickinson, USA) to analyze apoptosis.

### Small RNA Sequencing

Total RNA was qualified and quantified using a NanoDrop and an Agilent 2100 Bioanalyzer. Total RNA was purified by electrophoretic separation on a 15% urea denaturing PAGE gel, and regions corresponding to the 18–30-nt small RNA bands in the marker lane were excised and recovered. Then, the 18–30-nt small RNAs were ligated to a 5′-adaptor and a 3′-adaptor, which were subsequently transcribed into cDNA by SuperScript II Reverse Transcriptase (Invitrogen, USA), and then several rounds of PCR amplification with PCR Primer Cocktail and PCR Mix were performed to enrich the cDNA fragments. The PCR products were selected by agarose gel electrophoresis with target fragments of 100–120 bp and then purified with a QIAquick Gel Extraction Kit (QIAGEN, Valencia, CA). The final PCR ligation products were sequenced using the BGISEQ-500 platform (BGI-Shenzhen, China). Gene expression levels were measured in RPKM using Cufflinks.

### RNA Sequencing

RNA sample quality was analyzed, and cDNA libraries were synthesized and sequenced using BGI technology. In brief, an Agilent 2100 Bioanalyzer (Agilent) was used to assess the quality of the RNA samples and generate the cDNA libraries. Each library was sequenced on a HiSeq4000 (Illumina) using single reads. Gene expression levels were measured in RPKM using Cufflinks.

### Statistics

Data were analyzed using SPSS 13.0 (SPSS, Chicago, IL, USA) and reported as mean ± standard deviation (SD). Differences among the two groups were tested by Student's *t*-test or one-way ANOVA, as appropriate. *P* < 0.05 were considered significant. Diagrams were drawn using GraphPad Prism 7 software.

## Results

### The Expression of Hsa-miR-12462 Is Increased in AML Cases With Complete Remission (AML-CR) Over Cases With Relapsed/Refractory (AML-RR)

**Figure 1:**
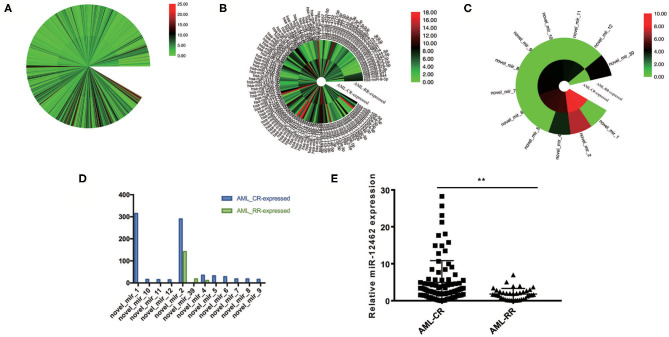
Identification of hsa-miR-12462 and its expression characteristics in acute myeloid leukemia (AML). **(A)** 1,099 differentially expressed miRNAs between patients who achieve complete remission (AML-CR) and patients who suffer relapse/refractory (AML-RR). **(B)** Two hundred fifty-one differentially expressed miRNAs showing over 2-fold significance between AML-CR cohort and AML-RR cohort. **(C)** Twelve unreported miRNAs among the 251 miRNAs. **(D)** novel_mir_1, which we named as hsa-miR-12462, showed the most significant difference. **(E)** Quantitative PCR analysis to compare the endogenous levels of hsa-miR-12462 in AML-CR and AML-RR patients (*p*-value: AML-CR vs. AML-RR < 0.01). ^**^p < 0.01.

To unveil the alternations between different curative efficacy, we collected 128 BM specimens composing of AML-CR (*n* = 90) and AML-RR (*n* = 38) and analyzed them by small RNA sequencing analysis. A total of 1,099 miRNAs displayed differences between the two cohorts (Figure 1A), and 251 miRNAs showed significant alternation >2-fold (Figure 1B). Among them, 12 miRNAs were unveiled for the first time (Figure 1C), and the most conspicuous one, hsa-miR-12462, demonstrated 315-fold increase in the AML-CR cohort greater than that in the AML-RR cohort (Figure 1D), as we previously verified (14). As to the tissue distribution of hsa-miR-12462 in mice, its level in skeletal muscle was significantly higher than that in lung, BM, kidney, heart, spleen, intestine, and stomach (14), while its expression in human and importance to mechanism need to be studied. In AML cell lines, its expression in HL-60 cells was higher than that in THP-1 cells (Supplementary Figure 1A). Next, 128 specimens were used to verify the differential expression of hsa-miR-12462 *via* quantitative RT-PCR. As anticipated, hsa-miR-12462 was confirmed to be upregulated in AML-CR patients vs. AML-RR patients (Figure 1E).

### FosB Is a Direct Target of Hsa-miR-12462

**Figure 2:**
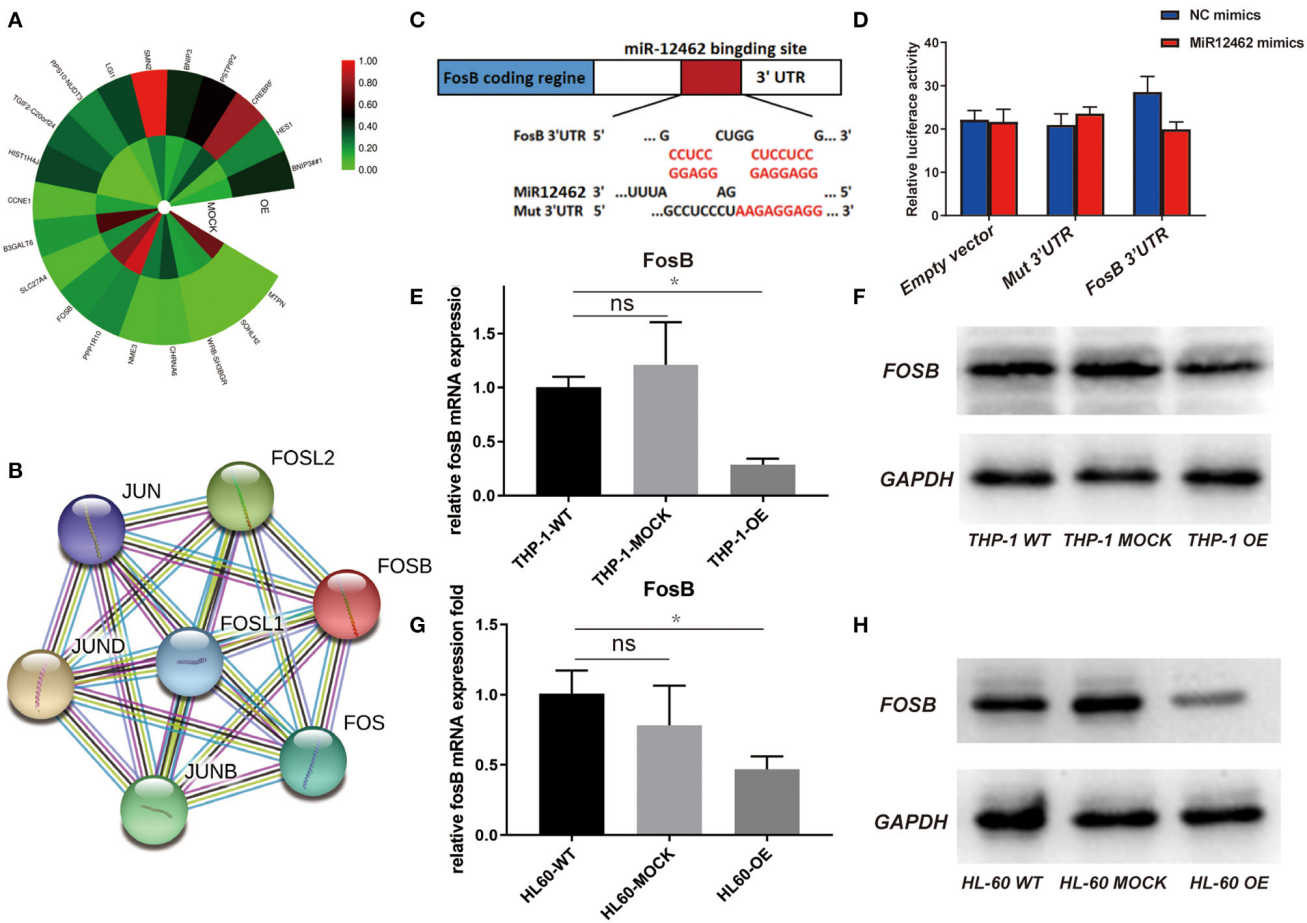
Direct regulation of hsa-miR-12462 on FosB expression. **(A)** RNA sequencing analysis to predict the target gene of hsa-miR-12462. OE represents hsa-miR-12462 overexpressed U937 cells, and MOCK represents mock-infected U937 cells. **(B)** Protein–protein interaction (PPI) network analysis to predict proteins interacted with FosB. **(C)** Online search program TargetScan to match the potential hsa-miR-12462 binding sites in FosB gene sequence. **(D)** Dual luciferase activity was assayed in hsa-miR-12462-mimic transfected and hsa-miR-12462 NC transfected 293T cells. Statistical analysis by Student's *t*-test. Data are mean ± SEM of three independent experiments (^*^p < 0.05). **(E,F)** Expression level of FosB in hsa-miR-12462-OE THP-1 cells, wild-type (WT) THP-1 cells, and MOCK THP-1 cells by quantitative PCR **(E)** and Western blotting **(F)** (*p*-value: THP-1 OE vs. THP-1 WT < 0.05). **(G,H)** Expression level of FosB in hsa-miR-12462-OE HL-60 cells, WT HL-60 cells, and MOCK HL-60 cells by quantitative PCR **(G)** and Western blotting **(H)** (*p*-value: HL-60 OE vs. HL-60 WT < 0.01). ^*^p < 0.05, ^**^p < 0.01.

To elucidate the detailed mechanisms of the suppressive effects of hsa-miR-12462 on phenotype changes, RNA sequencing (Figure 2A) together with protein–protein interaction (PPI) network analysis (Figure 2B) was performed in both hsa-miR-12462 overexpressed (OE) U937 cells and mock-infected (MOCK) U937 cells. We analyzed 307 differentially expressed genes in the hsa-miR-12462-OE group and discovered that the AP-1 transcription factor components including FosB were mostly involved. To clarify the interaction between hsa-miR-12462 and FosB, we firstly predicted the potential binding site for hsa-miR-12462 within the 3′-UTR region of FosB using the online search program TargetScan (Figure 2C). To prove the direct regulation of FosB by hsa-miR-12462, a mutant FosB at the putative binding sequence in a 3′-UTR element and a wild type (WT) were cloned into a dual luciferase reporter and then transiently transfected into 293T cells. Indeed, FosB mutation inhibited the hsa-miR-12462-mediated downregulation of luciferase activity (Figure 2D), indicating FosB was regulated by hsa-miR-12462 as a direct target.

To further corroborate these findings, we tested the expression of FosB both in THP-1 and HL-60 cell lines with different hsa-miR-12462 expressions (OE, WT, and MOCK). Overexpression of hsa-miR-12462 induced a statistically significant downregulation of FosB expression when compared with WT and MOCK groups, both at the mRNA (Figures 2E,G) and protein level (Figures 2F,H). Taken together, hsa-miR-12462 could suppress the expression of FosB in AML by direct interaction.

### The Expression of FosB Is Augmented in the AML-RR Group

**Figure 3:**
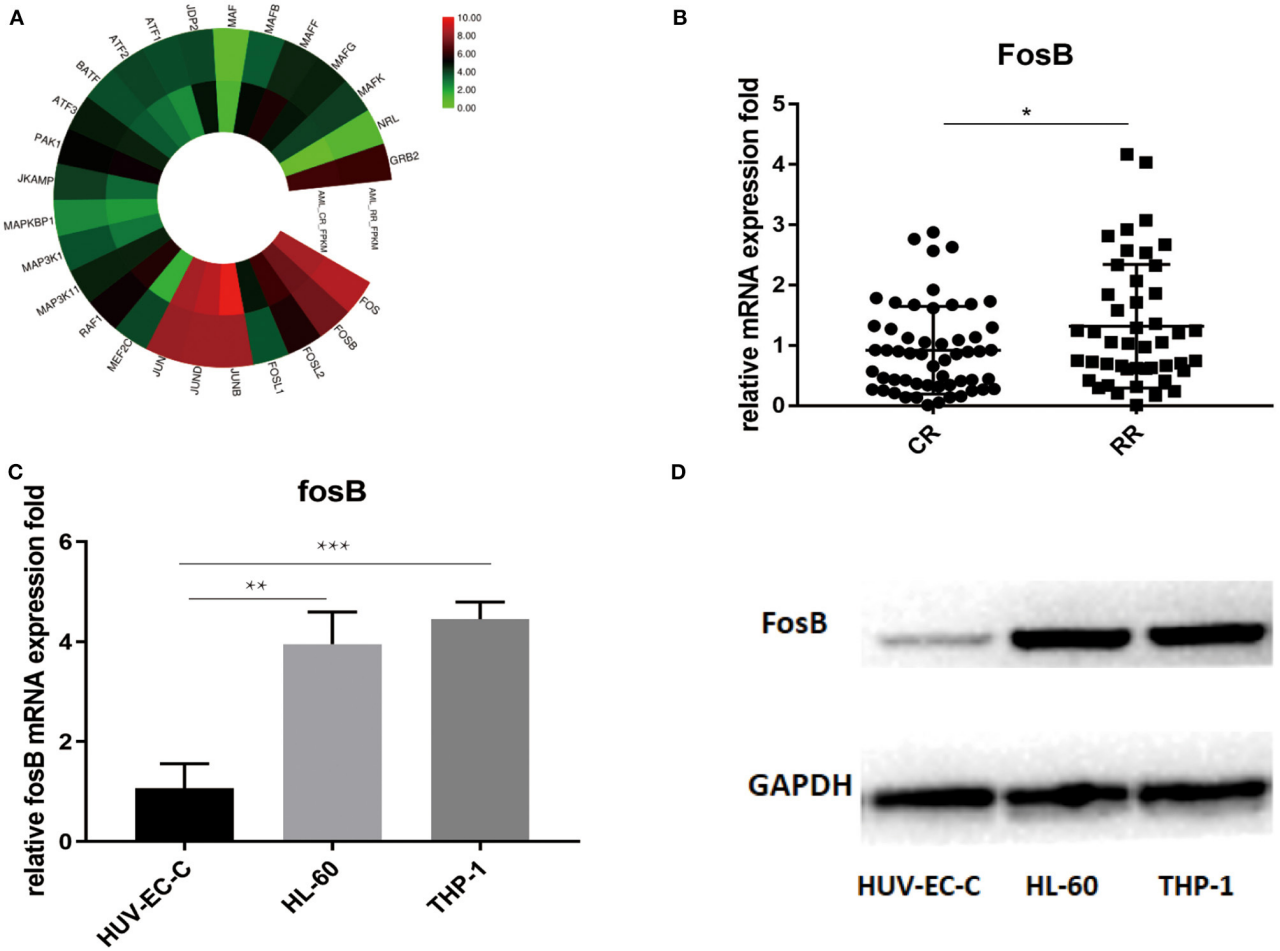
FosB is augmented in acute myeloid leukemia (AML) especially in AML-RR patients. **(A)** RNA sequencing analysis revealed the enhanced expression of activator protein (AP-1)-associated pathways in AML-RR vs. AML-CR. **(B)** Difference of FosB mRNA expression between AML-RR and AML-CR by quantitative PCR (*p*-value: CR vs. RR < 0.05). **(C)** FosB mRNA expression in normal human cell line (HUV-EC-C) and AML cell lines (HL-60 and THP-1) by quantitative PCR (*p*-value: HL-60 vs. HUV-EC-C < 0.01, THP-1 vs. HUV-EC-C < 0.001). **(D)** FosB protein level in normal human cell line (HUV-EC-C) and AML cell lines (HL-60 and THP-1) by Western blotting. ^*^p < 0.05, ^**^p < 0.01, ^***^p < 0.001.

As a key component of AP-1, FosB regulates gene networks associated with oncogenic transformation, reflecting in the proliferative and invasive modulation of solid tumor (15). Considering that the role of FosB to AML is still elusive, we aimed to depict more details on its specific expression and mechanism. RNA sequencing analysis revealed the distinctions on gene expression between AML-CR (*n* = 90) and AML-RR (*n* = 38). The results demonstrated that the expression of AP-1 components including FosB was augmented in the AML-RR group (Figure 3A, Supplementary Figure 1B). Next, the alternation of FosB was validated by quantitative RT-PCR among 128 patient samples. The results were in accordance with the RNA sequencing analysis, showing an apparent increase in the AML-RR cohort (Figure 3B). To identify whether the expression of FosB is AML-specific or widespread in normal tissues, we compared HUV-EC-C and AML cell lines (THP-1 and HL-60) of the FosB expression differences. FosB was more predominantly expressed in the AML than normal human cell lines, both at the mRNA (Figure 3C) and protein level (Figure 3D). Thus, we concluded that FosB was augmented in AML and more prominent in AML-RR vs. AML-CR patients and deduced that FosB might play an important role in modulating drug sensitivity and could be a potential therapeutic target.

### T-5224 Could Regulate the Proliferation, Apoptosis, and Drug Sensitivity in Acute Myeloid Leukemia Cells

**Figure 4:**
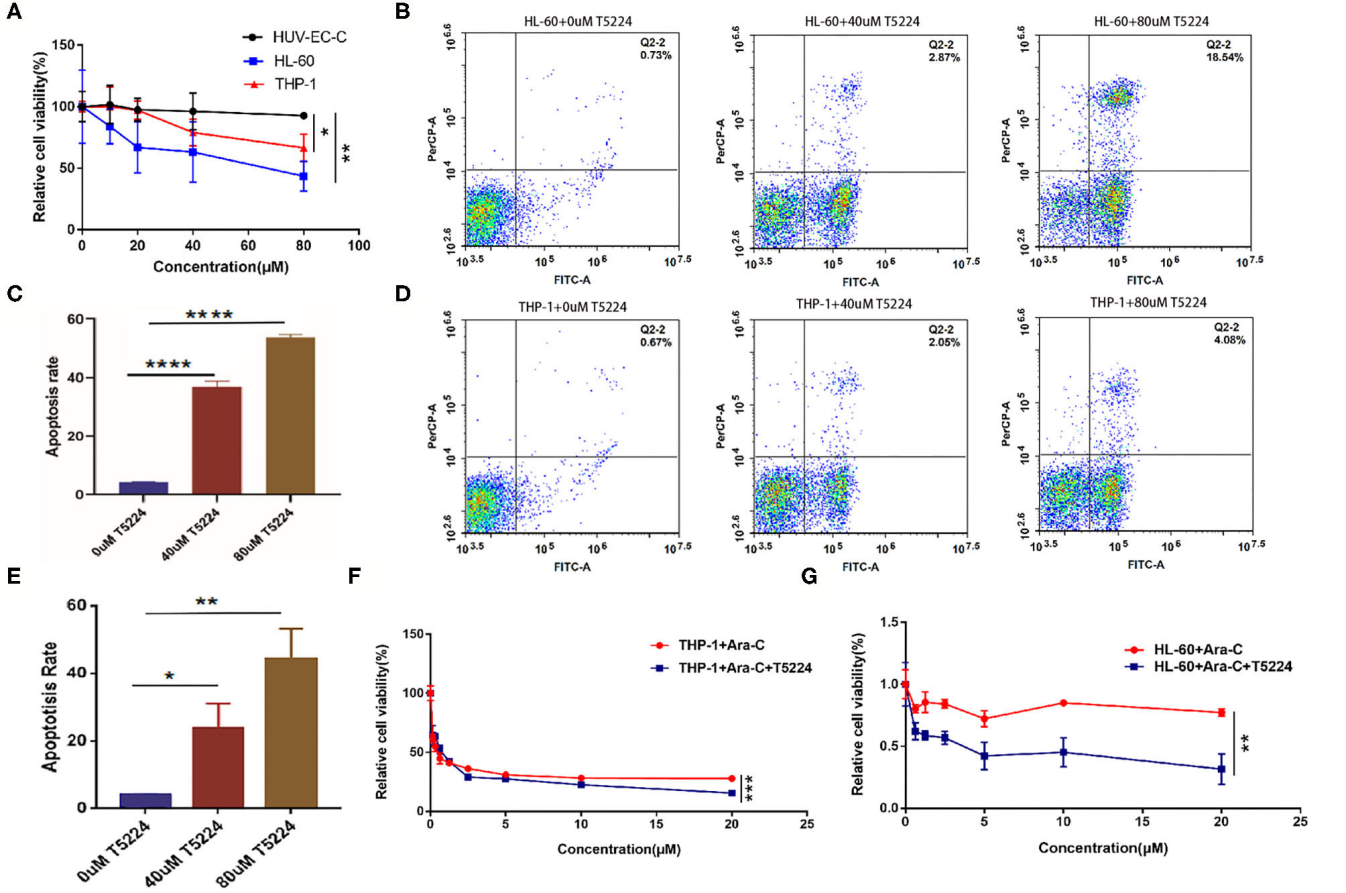
The function of T-5224 on the proliferation, apoptosis, and drug sensitivity in acute myeloid leukemia (AML) cell lines. **(A)** Cell proliferation of HUV-EC-C, HL-60, and THP-1 cells after T-5224 exposure for 24 h (*p*-value: THP-1 vs. HUV-EC-C < 0.05, HL-60 vs. HUV-EC-C < 0.01). **(B,C)** Flow cytometry analysis **(B)** and relative apoptosis rate **(C)** of HL-60 cell lines after being treated with different concentrations of T5224 for 24 h (*p*-value: HL-60 + 0 μM T-5224 vs. HL-60 + 40 μM T-5224 < 0.0001; HL-60 + 0 μM T-5224 vs. HL-60 + 80 μM T-5224 < 0.0001). **(D,E)** Flow cytometry analysis **(D)** and relative apoptosis rate **(E)** of THP-1 cell lines after being treated with different concentrations of T5224 for 24 h (*p*-value: THP-1 + 0 μM T-5224 vs. THP-1 + 40 μM T-5224 < 0.05, THP-1 + 0 μM T-5224 vs. THP-1 + 80 μM T-5224 < 0.01). **(F,G)** Cell viability of THP-1 cells **(F)** and HL-60 cells **(G)** after cotreatment with Ara-C ± T-5224 (*p*-value: THP-1 + Ara-C vs. THP-1 + Ara-C + T-5224 < 0.001, HL-60 + Ara-C vs. HL-60 + Ara-C + T-5224 < 0.01). ^*^p < 0.05, ^**^p < 0.01, ^***^p < 0.001, ^****^p < 0.0001.

To delineate the concrete role of FosB in AML, we next depleted the function of FosB by pharmacological inhibition. T-5224 is a non-peptidic small molecule designed as an AP-1-specific inhibitor. It can inhibit the binding of heterodimer AP-1 formed by FosB and Jun protein to AP-1 binding site in the promoter region, thus inhibiting the activation of AP-1 signaling pathway (16). HL-60, THP-1, and HUV-EC-C cells were inoculated with T-5224 at a concentration gradient and were detected with CCK8 assay after 24 h. Cell proliferation was significantly suppressed in AML cell lines while almost unaffected in HUV-EC-C cells (Figure 4A). Moreover, coincident with what we discovered above that miR-12462 reduced the expression of FosB in AML, T-5224 exerted little influence on viability in hsa-miR-12462-OE cells (Supplementary Figure 1C). Furthermore, we aimed to elaborate the effect of T-5224 on apoptosis. After 24 h exposure of T-5224, the percentage of apoptotic cells elevated, accompanied by progressively increased concentration (Figures 4B–E).

Ara-C is widely administrated in clinical practice as an indispensable component of induction and consolidation therapy. We treated AML cells with Ara-C ± T-5224 (40 μM) to illustrate the collaboration between Ara-C and T-5224. The results showed that Ara-C combined with T-5224 exhibited a stronger inhibitory effect on cell proliferation than monotherapy (Figures 4F,G). These findings indicated FosB might be related to the sensitivity of AML cells to chemical drugs.

## Discussion

Over the past decade, the fields of applied and functional miRNA research have been explored and miRNAs have been implicated across a spectrum of diseases as biomarkers, therapeutics, or vital regulators (17). As to AML, miRNAs may counteract with oncogenes or tumor suppressors to modulate the development and differentiation of hematopoietic cells, thereby contributing to AML formation (18). Our small RNA sequencing analysis revealed the miRNA profiling signatures between AML-CR and AML-RR patients. We chose hsa-miR-12462, which presents the most obvious difference, as we verified before. In clinical BM samples, the expression profile of has-miR-12462 corresponded with our previous findings, showing significant increase in AML-CR patients vs. AML-RR patients. Next, we combined RNA sequencing, PPI network analysis, and bioinformatics method (TargetScan) to predict target genes. The results indicated that the key components of AP-1 complex were involved, and FosB might be the most likely target of hsa-miR-12462. Dual luciferase reporter assay was used for validation. Consistent with our expectations, overexpression of hsa-miR-12462 suppressed the level of FosB in both mRNA and protein aspects.

AP-1 has been implicated in lots of biological processes including cell proliferation, death, differentiation, and oncogenic transformation (19, 20). In our experiment, RNA sequencing analysis suggested that compared with the AML-CR group, the AML-RR group showed high expression of AP-1 components including FosB. It was in accordance with the quantitative RT-PCR results in clinical samples. The expression of FosB in AML cell lines (HL-60 and THP-1) significantly elevated compared with that in normal human cell line (HUV-EC-C). In addition, through comparing the results of proliferation and apoptosis of AML cell lines after intervention with an inhibitor of FosB, T-5224, it was demonstrated that FosB may play a pivotal role in modulating cell viability as well as death in AML and is related to sensitivity to Ara-C.

**Figure 5:**
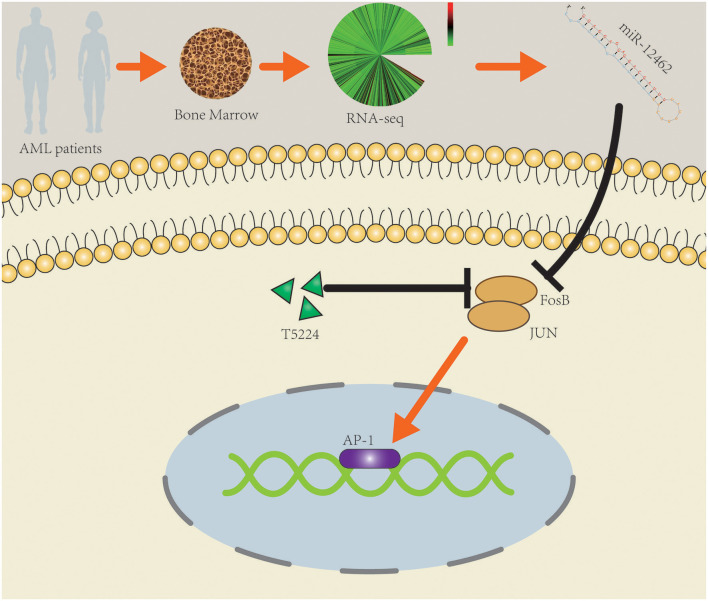
Schematic diagram of the potential roles of hsa-miR-12462 in modulating acute myeloid leukemia (AML) cell proliferation and drug sensitivity by targeting FosB.

In conclusion, the expression of hsa-miR-12462 was augmented significantly in AML-CR patients. Upregulation of hsa-miR-12462 influenced the proliferation and apoptosis of AML cell lines through negatively targeting FosB. Combining T-5224 and Ara-C had better cytotoxic impact than monotherapy. The findings provide new evidences about the underlying molecular mechanisms of hsa-miR-12462 and FosB (Figure 5), which deserves exploration in more detail through *in vivo* studies.

## Data Availability Statement

The dataset presented in this study can be found in Gene Expression Omnibus (GEO) repository. The accession number of the repository is GSE156654.

## Ethics Statement

The studies involving human participants were reviewed and approved by the Medical Institutional Ethics Committee of the Xiangya Hospital of Central South University. Written informed consent to participate in this study was provided by the participants' legal guardian/next of kin.

## Author Contributions

HZe designed the project and approved the final version. HW, HZh, LJ, TZ, SX, YJ, WL, and HL performed the experiments. HW and HZh analyzed the data. HW, XJ, and HZh wrote the manuscript. All authors contributed to the article and approved the submitted version.

## Conflict of Interest

The authors declare that the research was conducted in the absence of any commercial or financial relationships that could be construed as a potential conflict of interest.

## Supplementary Material

Supplementary Figure 1 **(A)** The expression of hsa-miR-12462 in HL-60 and THP-1 cell lines. **(B)** The expression of FosB is augmented in AML-RR group. **(C)** The exposure of T-5224 shows no influence on viability in hsa-miR-12462-OE HL-60 cells.
