# Supplementary figures and images for: A Novel miRNA Restores the Chemosensitivity of AML Cells Through Targeting FosB
Source: Front Med (Lausanne). 2020 Oct 6;7:582923. doi: 10.3389/fmed.2020.582923 (PMC7573296; doi:10.3389/fmed.2020.582923)

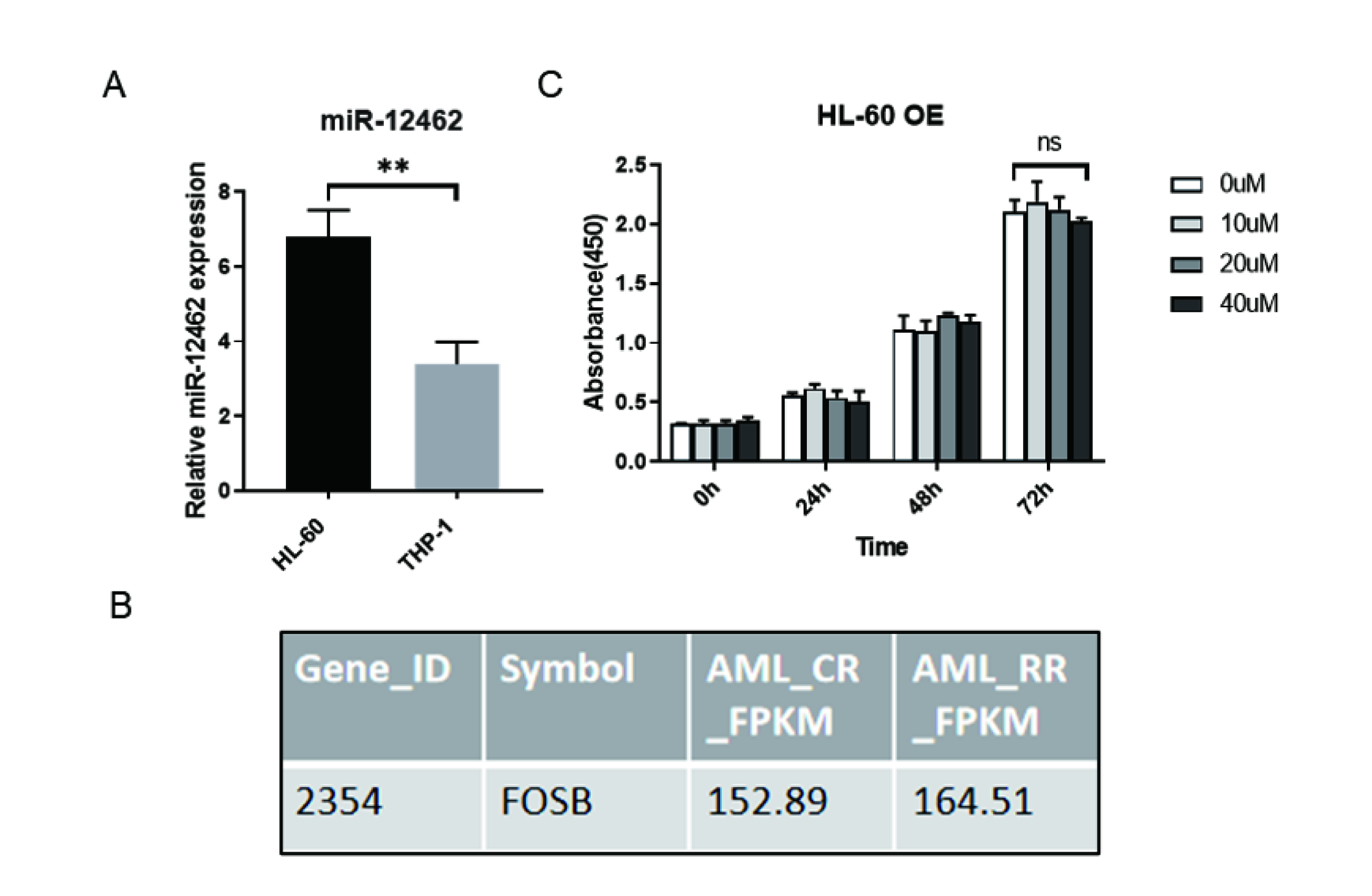

Supplement: Supplementary Figure 1 — (A) The expression of hsa-miR-12462 in HL-60 and THP-1 cell lines. (B) The expression of FosB is augmented in AML-RR group. (C) The exposure of T-5224 shows no influence on viability in hsa-miR-12462-OE HL-60 cells. [file Image_1.TIF]
